# “Huoling Shengji granule” for amyotrophic lateral sclerosis: protocol for a multicenter, randomized, double-blind, riluzole parallel controlled clinical trial

**DOI:** 10.3389/fnagi.2023.1153973

**Published:** 2023-05-09

**Authors:** Xiaolu Liu, Tingting Qin, Tao Li, Lei Shan, Xiang Lei, Xin Xu, Bin Wen, Yi Feng, Ping Yin, Dongsheng Fan

**Affiliations:** ^1^Department of Neurology, Peking University Third Hospital, Beijing, China; ^2^Beijing Municipal Key Laboratory of Biomarker and Translational Research in Neurodegenerative Diseases, Beijing, China; ^3^Key Laboratory for Neuroscience, National Health Commission/Ministry of Education, Peking University, Beijing, China; ^4^Department of Epidemiology and Biostatistics, School of Public Health, Tongji Medical College of Huazhong University of Science and Technology, Wuhan, China; ^5^Xiyuan Hospital, China Academy of Chinese Medical Sciences, Beijing, China; ^6^Foshan Kaichuan Pharmaceuticals Co., Ltd., Foshan, China; ^7^Beijing Qi-Huang Technology Co., Ltd., Beijing, China; ^8^Shanghai Pharma Rare Disease Medicine Co., Ltd., Shanghai, China; ^9^Engineering Research Center of Modern Preparation Technology of TCM, Shanghai University of Traditional Chinese Medicine, Shanghai, China

**Keywords:** amyotrophic lateral sclerosis, randomized controlled trial, traditional Chinese medicine, Huoling Shengji granule, flaccidity syndrome

## Abstract

**Background:**

There is still a large demand for effective treatments to delay disease deterioration in amyotrophic lateral sclerosis (ALS). Typical symptoms of ALS are considered “flaccidity syndrome” in traditional Chinese medicine (TCM). Huoling Shengji Granule (HLSJ) is a TCM formula used to treat flaccidity syndrome. Results of preclinical tests and a previous clinical study support HLSJ as a novel drug for ALS patients. This trial proposed to examine whether a 48-week course of HLSJ is effective and safe for ALS patients diagnosed with the Chinese medicine syndrome of spleen qi insufficiency and kidney yang deficiency.

**Methods and analysis:**

In this phase II, multicenter, randomized, double-blind, riluzole parallel-controlled, superiority-design study, eligible participants had the equal opportunity to be assigned to receive either HLSJ or riluzole randomly. Eleven specialized ALS centers in Mainland China will recruit 144 patients for this trial. The primary and secondary outcomes included the change in the ALSFRS-R score and the Rasch-Built Overall Amyotrophic Lateral Sclerosis Disability Scale (ROADS) from baseline to Week 48.

**Discussion:**

Here, we endeavored to evaluate TCM for ALS using a standard evidence-based approach for the first time. In addition, the ROADS, a self-report linear-weighted questionnaire, was selected as a secondary outcome measure. We expect to offer a new reference for the outcome evaluation of ALS trials.

**Clinical trial registration:**http://www.Chictr.org.cn, identifier ChiCTR2100044085.

## 1. Introduction

Up to now, only three interventions have received approval for the treatment of amyotrophic lateral sclerosis (ALS). Survival is prolonged by approximately 3 months with treatment of riluzole (Miller et al., 2012), and intravenous edaravone has been reported to be effective in slowing disease progression in a well-defined population in Japan (Writing Group, Edaravone (MCI-186) ALS 19 Study Group, 2017); however, the latter has not been verified in a real-world study in Europe (Witzel et al., 2022). AMX0035 increased the median survival by 6.5 months in a phase 2 trial (Paganoni et al., 2020). However, considering that the median survival in ALS is only 2–4 years (Tang and Fan, 2022), there is still a large demand to find more effective treatments to decelerate disease progression and prolong survival.

For ALS patients, the typical symptoms are muscle weakness, muscle atrophy, and difficulty in speaking or swallowing, which are considered “flaccidity syndrome” or “Wei Zheng” in traditional Chinese medicine (TCM). Based on the TCM theory, flaccidity syndrome is mainly induced by deficiency syndrome of the lung, spleen, liver, and kidney. Therefore, invigorating the spleen, replenishing qi, and nourishing the liver and kidney are considered as the principles of treatment, treating both the symptoms and root cause of disease (Zhu et al., 2017; Pan et al., 2021).

Huoling Shengji Granule (HLSJ) is a TCM formula for the treatment of Wei Zheng by tonifying qi, strengthening the spleen, warming the kidney, and invigorating yang. It consists of six herbs, with the proportion prescribed in a previous study (Radix Astragali: Epimedium herb: Radix Rehmanniae: Fructus Corni: Atractylodes macrocephala Koidz: Poria cocos = 6:5:5:4:3:3; Wu et al., 2021). The monarch herb, Radix Astragali, is used to invigorate middle-jiao energy and replenish qi; Epimedium herb is chosen as the monarch herb to unchoke the Governor Vessel and warm kidney-yang; as a minister herb of Radix Astragali, *Atractylodes macrocephala* Koidz facilitates tonifying qi of the stomach and spleen; Fructus Corni, the other minister herb of Epimedium Herb, promotes tonifying the liver, stomach, and kidney; *Poria cocos* can help tonify qi of the spleen without causing stagnation; and the cool nature of Radix Rehmanniae can regulate the heat property of the whole formula by nourishing yin (Zhou et al., 2018).

In the preclinical study, medium-dose HLSJ prolonged the mean survival and disease course of SOD1^G93A^ mice compared with the vehicle group with statistical significance, while the disease onset was not significantly delayed. In HLSJ group, motor neuron loss was prevented in the lumbar spinal cords in Nissl and TUNEL staining, and the aberrant expression of apoptosis-related proteins was corrected. In addition, HLSJ relieved atrophy of the gastrocnemius muscle and loss of the neuromuscular junction. HLSJ treatment also inhibited glial cell activation and suppressed the inflammatory response. Based on the above results, HLSJ has shown therapeutic effects in the animal model of ALS, which indicates HLSJ as a potential novel drug for ALS patients (Zhou et al., 2018).

In a previous clinical study, 64 patients with ALS were included and randomly divided into HLSJ decoction and riluzole groups, with a course of 12 weeks. No statistical differences were detected in changes in the Advanced Norris Scale score between the two groups. Compared to riluzole, the HLSJ decoction effectively decreased the symptom scores of the Chinese medicine syndrome. In consequence, HLSJ is worth further investigation as a promising drug for ALS (Sui et al., 2016).

The current phase II, multicenter, randomized, double-blind, riluzole parallel-controlled, superiority-design study is proposed to verify the efficacy and safety of HLSJ as a treatment for a 48-week period in ALS patients diagnosed with Chinese medicine syndrome of insufficiency of spleen qi and deficiency of kidney yang, compared with treatment with riluzole. Eligible participants had the equal opportunity to be assigned to receive either HLSJ or riluzole randomly.

## 2. Methods and analysis

### 2.1. Study setting

Eleven specialized ALS centers in Mainland China will recruit patients for this trial. All centers are members of the ALS Collaborative Group in China, awarded national or regional centers for the diagnosis and treatment of ALS, and all are able to conduct subject recruitment, management, and follow-up. Detailed information on the ALS centers, independent data monitoring committee, statistics units, contract research organization (CRO), site management organization, and sponsors is shown in Table 1.

**Table 1:** Institutions involved in the study.

Institution	City	Role and activity	Work package
Peking University Third Hospital	Beijing	Coordinating center	Convene expert committee, formulate the protocol and create the case report form (CRF) with statistics unit.
			Submit application to ethical committees.
			Train the protocol and outcomes measure.
			Present and disseminate the results with the permission of the sponsor.
			Enroll and follow-up the subjects.
The Second Hospital of Hebei Medical University	Shijiazhuang	Enrolling center	Participate in the seminar on protocol formulation and provide suggestions.
Henan Provinclal People’s Hosipital	Zhengzhou		Complete the training of the protocol and outcomes measure.
The First Affiliated Hosipital, Sun Yat-Sen University	Guangzhou		
			Enroll and follow-up at least four subjects.
Guangdong Province Traditional Chinese Medical Hospital	Guangzhou		
The Second Affiliated Hospital, Zhejiang University School of Medicine	Hangzhou		
West China Hospital of Sichuan University	Chengdu		
The First Affiliated Hospital of Xi’an Jiaotong University	Xi’an		
Fujian Medical University Union Hospital	Fuzhou		
The First Affiliated Hospital of Harbin Medical University	Harbin		
Renmin Hospital of Wuhan University	Wuhan		
/	/	Independent Data Monitoring Committee	Safety monitoring, efficacy monitoring, operational quality control, and suggestions for adjustment of trial design.
			Conduct an interim analysis and assess the risks and benefits for the subjects.
			Make a key recommendation whether the trial continues.
Wuhan Zhizhi Medical and Pharmaceutical Technology Co., Ltd.	Wuhan	Statistics unit	Participate in the design of the protocol and statistical plans.
			Establishment and management of Electronic Data Capture System and central randomization system.
			Blind codes.
			Statistical analysis.
Beijing Qi-Huang Technology Co., Ltd.	Beijing	Contract Research Organization	Monitor the trial.
Foshan Kaichuan Pharma Co., Ltd., Shanghai Pharma Rare Disease Medicine Co., Ltd	Foshan Shanghai	sponsor and funders	Provide funding.
			Provide HLSJ, riluzole tablets and simulants.
			Present and disseminate the results.
Medkey Med-Tech Development Co., Ltd.	Shanghai	Site Management Organization	Assist investigators in completing the screening, enrolling, and follow-up of the subjects.

### 2.2. Participants

The trial will enroll patients diagnosed with ALS according to the revised El Escorial criteria (Brooks et al., 2000) and the criteria of insufficiency of spleen qi and deficiency of kidney yang in TCM, which must be evaluated by the TCM doctor in the enrolling center. For the criteria of insufficiency of spleen qi and deficiency of kidney yang in TCM, the main symptoms include weakness, atrophy, and fasciculations of the muscles. Spiritual exhaustion, shortness of breath, fear of cold or limb cooling, spontaneous sweating, and weakness of the waist and knees were considered minor symptoms. The tongue is reddish or light and the pulse is heavy and thin. Patients with at least two main symptoms and at least two minor symptoms, along with the corresponding condition of the tongue and pulse, can be diagnosed with insufficiency of spleen qi and deficiency of kidney yang in TCM.

### 2.3. Eligibility criteria

#### 2.3.1. Inclusion criteria

1. Patients diagnosed with clinically definite ALS, clinically probable ALS, or clinically probable-laboratory-supported ALS.
2. Each term of the ALS Functional Rating Scale Revised (ALSFRS-R) score is no less than two points (dyspnea, orthopnea, and respiratory insufficiency must be 4 points).
3. Forced vital capacity (FVC) is no less than 70% of the predicted value for a person’s age, sex, and height.
4. Disease duration ≤3 years at the screening visit.
5. TCM syndrome of insufficiency of spleen qi and deficiency of kidney yang.
6. Aged 45–70 years, male or female.
7. Patients who voluntarily signed an informed consent (IC) form.

#### 2.3.2. Exclusion criteria

1. Patients diagnosed with familial ALS.
2. Presence of gastrostomy.
3. The presence of other neurological diseases that mimic ALS or disturb the evaluation of its efficacy, such as cervical spondylosis myelopathy, lumbar spondylosis, and dementia.
4. Patients with a conduction block in motor nerves or abnormalities in sensory nerves on electromyography or with lesions in images (magnetic resonance imaging or computed tomography) that might explain the clinical findings.
5. Exposure to riluzole or edaravone within 3 months of screening. If previously exposed to riluzole or edaravone, a 3-month washout period will be required prior to screening.
6. A spinal operation after symptom onset of ALS.
7. Alanine aminotransferase (ALT) and/or aspartate aminotransferase (AST) exceeds 1.5 times the upper limit of normal (ULN). Serum creatinine exceeds the ULN.
8. Any other clinically significant neurological, cardiac, pulmonary, hematological, endocrine, or psychiatric disorders.
9. History of alcohol or drug abuse.
10. Pregnant women or women currently breastfeeding. Women of reproductive age refuse an effective conception control from screening to 3 months after the day of the last dose.
11. History of a known allergy to the drugs or the excipients involved in the current trial.
12. Within 3 months prior to screening, enrollment in other clinical trials.

### 2.4. Recruitment

Recruitment was conducted by the principal investigator or sub investigators at each enrolling center. Information about the current trial was distributed through recruitment notices, which was approved by the Ethics Committee. Subjects signed the IC voluntarily only after the investigators fully explained the procedures and disclosed possible benefits, potential risks, and the contact information of the investigator of the trial.

### 2.5. Randomization

In this study, the block randomization was adopted. The randomization lists were generated by an independent statistician using SAS V9.4 and then introduced into the central randomization system (Interactive web response system, IWRS). Patients had the equal opportunity to be assigned to receive either HLSJ or riluzole randomly, with no stratification. All patients with the IC signed received a unique identification number (ID) through the IWRS at the screening visit. This number will be constant throughout the entire study. The ID list of patients was linked to the treatment code labeled on the investigational medicinal product (IMP) boxes.

### 2.6. Blinding procedures

The current trial was designed as a double-blind (participant- and investigator-blind) treatment. The similarity of HLSJ and its placebo in terms of both granules and solution status was proven in a previous study (Wu et al., 2021). Riluzole and placebo were indistinguishable in appearance and packaging. IMP is provided by the sponsor and then packaged and labeled by authorized people not participating in the observation, monitoring, and statistical analysis.

The randomization lists and parameters for randomization, such as the random seed and block length, are sealed and kept safe by the principal investigator and sponsor. A code revealing the assignment for a specific subject cannot be opened during the study unless the subject’s therapy assignment is necessary for the choice of treatment. The patient will withdraw from further participation in the study if his code is broken. One exception is that in the event of suspected unexpected serious adverse reactions (SUSAR), the Pharmacovigilance Division may unilaterally unblind a single case according to the submission requirements of the administration, with all others remaining blind. The SUSAR subject will not be considered to have dropped out.

### 2.7. Intervention and procedure

In the HLSJ group, the trial medication will be supplied as one bag of HLSJ (20 g per bag) and one tablet of placebo riluzole twice daily. In the control group, the subjects will receive one bag of placebo HLSJ and one tablet of riluzole (50 mg per tablet) twice daily. The HLSJ or placebo granule and one cup of room temperature water should be mixed and stirred vigorously, and then drunk completely within 1 h of the brew. The treatment will be performed for 48 weeks.

**Figure 1:**
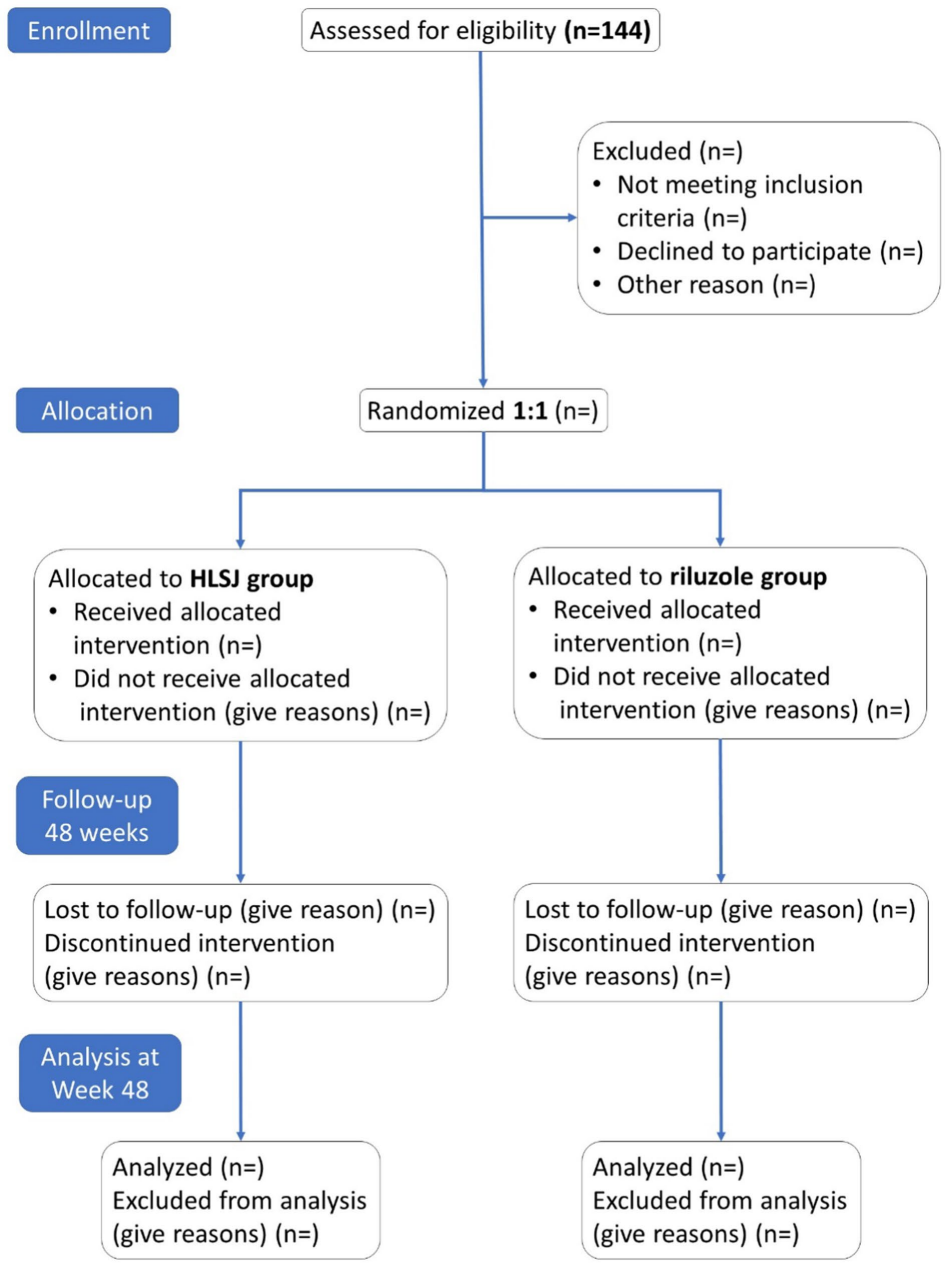
Study workflow.

The trial will consist of 14 visits (V0–V13). The screening visit will be carried out within 7 days before the first dose treatment. Subsequently, the participants will be observed every 12 weeks at five site-visits, V1, V4, V7, V10, and V13. A phone call visit will be performed every 4 weeks during the interval between site visits. The detailed trial procedure and workflow are presented in Figure 1 and Table 2.

**Table 2:** Study flow chart.

	V0	V1	V2	V3	V4	V5	V6	V7	V8	V9	V10	V11	V12	V13
	-7d ~ 0d	W0	W4 ± 3d	W8 ± 3d	W12 ± 7d	W16 ± 3d	W20 ± 3d	W24 ± 7d	W28 ± 3d	W32 ± 3d	W36 ± 7d	W40 ± 3d	W44 ± 3d	W48 ± 7d
	Screening	Baseline	phone call	phone call	visit	phone call	phone call	visit	phone call	phone call	visit	phone call	phone call	visit
Patient information
Informed consent	X													
Demographics	X													
Medical history	X													
TCM syndrome differentiation	X													
Concomitant treatment history	X		X	X	X	X	X	X	X	X	X	X	X	X
Screening assessment
Pregnancy test in fertile women	X													
Neuro-electrophysiological examination	X													
Inclusion/exclusion criteria	X													
Randomization		X												
Efficacy outcomes
ALSFRS-R	X	X			X			X			X			X
ROADS		X			X			X			X			X
ALSAQ-40		X			X			X			X			X
Forced vital capacity	X	X			X			X			X			X
SSCMS		X			X			X			X			X
Endpoint event					X			X			X			X
Safety outcomes
Adverse events			X	X	X	X	X	X	X	X	X	X	X	X
Vital signs and physical examination	X	X			X			X			X			X
Laboratory tests	X				X			X			X			X
Electrocardiography	X				X			X			X			X
Others
Study treatment dispensation		X			X			X			X			
Study treatment compliance					X			X			X			X

The coronavirus disease 2019 (COVID-19) pandemic is currently ongoing. If travel restrictions prevent the subjects from site visits, phone call visits or video visits are acceptable for the evaluation of ALSFRS-R and symptom scores of Chinese medicine syndrome (SSCMS; Table 3), with the source files of the process saved. Safety laboratory tests, electrocardiograms, and pulmonary function tests can be completed at a local center according to the patient’s convenience. Photos of results will be sent to the corresponding investigator to review, evaluate, and interpret the results in time. IMP, the remaining drugs, and patient diaries will be delivered by post.

**Table 3:** The symptom scores of Chinese medicine syndrome.

The symptom scores of Chinese medicine syndrome (SSCMS)				
*Main symptoms*	*None (0)*	*Mild (2)*	*Moderate (4)*	*Severe (6)*
Weakness	None	Slightly worse than normal	Slightly inconvenient in daily life	Difficult to hold objects and walk
Atrophy	None	Mild atrophy in the first interosseous muscle, with hand function not affected	Obvious atrophy in the first interosseous muscle, with hand function significantly affected	Significant atrophy in the first interosseous muscle, difficult to hold objects
Fasciculation	None	Local and occasional	Multifocal and frequent	Diffuse and persistent
*Minor symptoms*	*None (0)*	*Mild (1)*	*Moderate (2)*	*Severe (3)*
Spiritual exhaustion	None	Feel tired about oneself	Others can feel someone tired	Too fatigue to keep talking
Shortness of breath	None	Occurs when active	Occurs after slight activity	Occurs at rest
Fear of cold or limb cooling	None	Slight	Obvious	Significant: needs more clothes and bedding
Spontaneous sweating	None	Slight and occasional, clothes keep dry	Obvious, clothes wet frequently	Significant: need to change clothes daily
Weakness of waist and knees	None	Occasional	Frequent	Too significant to walk

Total SSCMS: 0–33.

ΔSSCMS = [(SSCMS before treatment-SSCMS after treatment)/SSCMS before treatment]*100%.

Significantly effective: ΔSSCMS no less than 70%.

Effective: ΔSSCMS no less than 30%, but less than 70%.

Ineffective: ΔSSCMS less than 30%.

### 2.8. Concomitant medications

All non-IMP medications taken at the initiation of the study or given additionally during the study should be recorded as concomitant medications. It is permitted to continue treatment for other medical conditions with no effect on the evaluation of the current trial. Supportive treatments, such as nutritional management and respiratory support, are recommended according to clinical guidelines if the situation deteriorates during the study. In view of the warm nature of IMP, TCM drugs that nourish yin are permitted when patients experience night sweats or constipation. Adequate treatment is recommended in the case of adverse events. It is permitted that TCM drugs with similar indications and functions are taken continuously for no more than 1 month, with a total course of no more than 2 months. The details of any other treatment, including the reason for use, name, dosage, and duration of the drugs, should be recorded in the source documents and documented in the electronic case report form (eCRF).

Riluzole (not IMP), edaravone, gene therapy, and stem cell therapy are prohibited throughout the study.

### 2.9. Outcomes

#### 2.9.1. Primary outcome

The primary outcome was the change in ALSFRS-R scores from baseline to Week 48.

#### 2.9.2. Secondary outcomes

The secondary outcomes of the study include the following:

1. Changes in ALSFRS-R from baseline to Week 12, 24, and 36.
2. Changes in Rasch-Built Overall Amyotrophic Lateral Sclerosis Disability Scale (ROADS) scores from baseline to Week 12, 24, 36, and 48.
3. Changes in the percentages of measured to predicted values of FVC from baseline to Week 12, 24, 36, and 48.
4. Changes in 40-item Amyotrophic Lateral Sclerosis Assessment Questionnaire (ALSAQ-40) scores from baseline to Week 12, 24, 36, and 48.
5. Changes in the SSCMS from baseline to Week 12, 24, 36, and 48, and the efficacy of Chinese medicine syndrome.
6. The rate of endpoint events and the time to endpoint events were defined as death, tracheostomy, or permanent-assisted ventilation (>22 h daily for >14 days).

#### 2.9.3. Safety

All adverse events (AE), irrespective of their seriousness, will be collected and reported. Severity of AE is graded according to Common Terminology Criteria for Adverse Events Version 5.0. The relationship between the AE and IMP is evaluated by the investigators. Once an AE is detected, the subject should be carefully evaluated, treated in a timely manner, and followed continuously until the condition is stable. SUSAR will be promptly reported to all investigators, clinical trial institutions, and ethics committees by the sponsor. The sponsor also has a duty to report SUSAR to the Medical Products Administration and Health Administrative Department. For life-threatening events, the interval should not exceed 7 days. Other events should be reported within 15 days.

### 2.10. Statistical consideration

#### 2.10.1. Sample size calculation

The superiority test was performed for the comparison of the primary outcome in the two groups. Based on a previous real-world study of the long-term use of riluzole (Chen et al., 2016) and the consensus of the experts involved in the current study, we assumed that, for the changes of ALSFRS-R score, the adjusted mean superiority through 48 weeks between the HLSJ group and control group was 2.0 points, with a standard deviation of 4.0 points. Therefore, we needed to include 64 patients in each group (128 patients in total) to provide 80% statistical power with an alpha level of 0.05 in a two-sided test. Assuming an average dropout rate of 10%, recruitment of 144 patients will be necessary.

#### 2.10.2. Analytic populations

All randomized participants, who take at least one dose of IMP and have at least one evaluation of therapeutic efficiency post baseline, are defined as the full analysis set for the primary analysis. Patients with serious protocol deviations are further excluded in a prespecified secondary analysis of the per-protocol population. Patients receiving IMP with a safety assessment will be included in the safety analysis.

The full analysis population will be analyzed for baseline characteristics and efficacy evaluation. The secondary per-protocol analysis will be performed for efficacy assessment only, and the safety analysis will be conducted only to evaluate safety.

#### 2.10.3. Statistical analyzes

The data analysis followed the principle of intention-to-treat (ITT). The primary outcome will be compared between the two arms using the analysis of covariance for adjusted analysis, with ALSFRS-R at baseline as the covariate in the model and HLSJ group as the fixed effect. The least squares mean (LSMEAN) and its 95% confidence interval (CI) will be measured. If the lower limit of the 95% CI of the LSMEAN difference between the HLSJ and riluzole group is greater than zero, the HLSJ group will be considered superior to the riluzole group. Intra-group changes from baseline will be tested using the Wilcoxon signed-rank test. Wilcoxon rank-sum test will be used in the test of between-group differences in changes.

A similar approach will be used to analyze the secondary outcomes. Wilcoxon rank sum test (ordered categorical data) or the Cochran–Mantel–Haenszel statistics with controlled central effects will be conducted between the two groups for categorical data. We also computed the risk difference (RD) and its 95% CI. For time-to-event data, the product-limit method will be used to calculate the quartiles and two-sided 95% CI of time to each endpoint event. The Kaplan–Meier curve will be constructed and analyzed using log-rank tests.

All safety data will be documented according to the System Organ Class and Preferred Term. Subgroup analyzes will be conducted according to clinical stratification (e.g., bulbar or limb onset). The last observation carried forward (LOCF) method will be performed to impute missing values of the primary outcome measure. Missing values for secondary outcomes and safety will not be processed with a similar approach. Once the database is locked, the statistical analysis plan will not be revised. Then, the statistical analysis will be performed using SAS V9.4. In all statistical analyzes, *p*-values <0.05 are considered statistically significant with two-sided test.

### 2.11. Withdrawal criteria

Consent withdrawal or loss to follow-up means that the subjects withdrew from the trial.

For some reason, the subject is no longer suitable for the current trial. The investigators have the discretion to withdraw the subject from the trial if the following conditions occur:

1. The health of the patient deteriorates or some serious complications occur.
2. Abnormal liver function with one of the following conditions present: (1) ALT or AST >8 times the ULN; (2) ALT or AST >5 times the ULN for 2 weeks; (3) ALT or AST >3 times the ULN, with total bilirubin >2 times the ULN or INR >1.5; (4) ALT or AST >3 times the ULN, with the following conditions: fever, rash, nausea, vomiting, severe fatigue, right upper abdominal pain, and/or percentage of eosinophilic granulocyte increased (>5%).
3. Neutrophile granulocyte in blood <2.0 × 10^9^/L.
4. Some special physiological situation, such as a female who becomes pregnant.
5. Allergy or serious adverse reaction.
6. Subjects unblinded during the trial (except SUSAR).
7. Other conditions, such as the subject refuses a necessary nasal feeding tube or gastrostomy.

### 2.12. Data recording and study monitoring

Electronic data capture was adopted for data entry, verification, storage, and management. The eCRF is designed according to the case report form approved by the Ethics Committee. Source documents, including the results of scales, laboratory tests, and signed IC forms will be safely stored in the hospital. Data in the eCRF must be in line with the source documents. All records will be in accordance with Good Clinical Practice (GCP) and relevant regulations in China.

The Independent Data Monitoring Committee (IDMC) consists of two experts in the field of clinician and safety and one statistical expert. They work independently and are not involved with sponsors and investigators. The responsibility of the IDMC includes safety monitoring, efficacy monitoring, operational quality control, and suggestions for adjustment of the trial design. They will conduct an interim analysis and assess the risks and benefits to the subjects. A key recommendation of “continue,” “continue after the protocol is adjusted,” or “terminate” will be made for the trial according to the interim analysis results. The sponsor decides whether to perform the IDMC recommendations.

The CRO is in charge of preparing essential documents for ethical review. The CRO organizes the initial meeting in each center to ensure that all the documents are well prepared, all the procedures of the trial are fully understood, and outcomes are measured consistently by the investigators. The CRO monitors and makes sure that the trial is processing in keeping with the protocol. The clinical research associate (CRA) should communicate with the investigators when a deviation from the protocol or nonstandard procedure occurs.

## 3. Discussion

In the real world, conventional medication cannot fully meet the needs of patients with ALS, and TCMs are used relatively widely. In a survey of 229 ALS patients from Shanghai, approximately 90% of patients reported a history of Chinese herbal decoctions or Chinese herbal compound treatment after the diagnosis of ALS (Pan et al., 2013). According to the TCM theory, invigorating the spleen and nourishing the kidney are considered as the principles of treatment. Some TCM formulations, formulated based on this principle, have shown potential efficacy (Zhu et al., 2017). Nevertheless, the level of evidence for these trials is insufficient because of the limited sample size, non-random or non-controlled design, inappropriate outcome measures, or lack of follow-up (Song et al., 2022). Here, we designed a randomized controlled trial (RCT) in patients with ALS to verify the efficacy and safety of HLSJ. This is the first attempt to evaluate TCMs for the treatment of ALS using a standard evidence-based approach.

The trial design and issues regarding methodology are possible reasons for the negative results in the previous RCTs (Mitsumoto et al., 2014). The ALSFRS-R and survival are the most common primary endpoint (Hashizume and Katsuno, 2022). However, the ALSFRS-R also has limitations that may influence the consequences of the trial. In the Pooled Resource Open-Access ALS Clinical Trials database, the ALSFRS-R score did not decline over 6 months in 25% of the participants. Fourteen percent of participants had reversals in their ALSFRS-R in a 180-day interval (Bedlack et al., 2016). This phenomenon is not consistent with the biological disease progression or the findings of objective outcome measures (Fournier et al., 2020). A previous Rasch analyzes indicated that ALSFRS-R was not linearly weighted and lacked unidimensionality (Franchignoni et al., 2013). The ROADS, a self-report linear-weighted questionnaire with ideal test–retest reliability, was considered a more sensible outcome measure than the ALSFRS-R in ALS clinical trials (Fournier et al., 2020). The Chinese version of the ROADS has also been verified on a linearly-weighted scale with proper validity and reliability (Sun et al., 2021). In the current trial, ROADS was selected as the secondary outcome measure. The advantages and reliability of the ROADS will be further verified in future RCTs.

## 4. Ethics and dissemination

All procedures conformed to the Declaration of Helsinki and GCPs. The trial protocol was registered in Chictr.org.cn (Chinese Clinical Trial Registry) as ChiCTR2100044085. All the documents, including the full protocol and IC forms, were approved by the 11 ethics committees. Updates to the study protocol should be approved by the Ethics Committee and promptly notified to the investigators. Only the investigators and CRAs involved in the current trial may have access to the personal medical records of the subjects. Clinical trial data will be collected and processed using hidden personal identification information. An insurance will cover the possible harm of subjects in the trial.

The results will be published in Chictr.org.cn and peer-reviewed academic journals, with the coordinating investigator as the corresponding author and other investigators as co-authors, after obtaining written approval from the sponsors. The metadata and study protocol will be shared within 6 months after the trial completion at http://www.chictr.org.cn. Then, the data can be searched and browsed by the public. Data can be downloaded from the sponsor on reasonable request.

## 5. Trial status

The general protocol in operation was Version 2.0, which took effect on November 7th, 2022. An additional contingency plan for the COVID-19 pandemic came into effect on November 3rd, 2021. The recruitment started in September 2021, enrollment was accomplished in May 2022, and all visits of the patients are scheduled to be completed in May 2023.

## Ethics statement

The studies involving human participants were reviewed and approved by Peking University Third Hospital Medical Science Research Ethics Committee. The patients/participants provided their written informed consent to participate in this study.

## Author contributions

XLiu: draft preparation and writing and editing. TQ: modify the data management and statistical analyzes section of the draft. TL: medical advisor to the draft. LS: draft preparation and funding acquisition. XLei, XX, and BW: draft preparation. YF: medical advisor to the draft. PY: review the data management and statistical analyzes. DF: conceptualization and supervision. All authors contributed to the article and approved the submitted version.

## Funding

This study received funding from Foshan Kaichuan Pharmaceuticals Co., Ltd. and Shanghai Pharma Rare Disease Medicine Co., Ltd. Foshan Kaichuan Pharma Co., Ltd. provided funding for medical writing support in the development of this protocol.

## Conflict of interest

LS was employed by Foshan Kaichuan Pharmaceuticals Co., Ltd. XLei was employed by Beijing Qi-Huang Technology Co., Ltd. XX and BW were employed by Shanghai Pharma Rare Disease Medicine Co., Ltd. The funder had the following involvement with the study, draft preparation and funding acquisition. Beijing Qi-Huang Technology Co., Ltd. is the contract research organization in the current trial.

The remaining authors declare that the research was conducted in the absence of any commercial or financial relationships that could be construed as a potential conflict of interest.

## Publisher’s note

All claims expressed in this article are solely those of the authors and do not necessarily represent those of their affiliated organizations, or those of the publisher, the editors and the reviewers. Any product that may be evaluated in this article, or claim that may be made by its manufacturer, is not guaranteed or endorsed by the publisher.

## Abbreviations

ALS: amyotrophic lateral sclerosis
ALSAQ-40: Amyotrophic Lateral Sclerosis Assessment Questionnaire
ALSFRS-R: ALS Functional Rating Scale Revised
CI: confidence interval
COVID-19: coronavirus disease 2019
eCRF: electronic case report form
FVC: forced vital capacity
GCP: Good Clinical Practice
HLSJ: Huoling Shengji Granule
IC: informed consent
IDMC: Independent Data Monitoring Committee
IMP: investigational medicinal product
LSMEAN: least squares mean
RD: risk difference
ROADS: Rasch-Built Overall Amyotrophic Lateral Sclerosis Disability Scale
SSCMS: symptom scores of Chinese medicine syndrome
SUSAR: suspected unexpected serious adverse reaction
TCM: traditional Chinese medicine
